# Exploring the interconnections of anxiety, depression, sleep problems and health-promoting lifestyles among Chinese university students: a comprehensive network approach

**DOI:** 10.3389/fpsyt.2024.1402680

**Published:** 2024-07-15

**Authors:** Changqing Sun, Zhengqi Zhu, Peijia Zhang, Lianke Wang, Qiang Zhang, Yuanli Guo, Lina Guo, Yang Li, Panpan Wang, Bo Hu, Mengting Liu, Jingyi Duan, Yiwen Wang, Ziqi Wang, Ying Qin

**Affiliations:** ^1^ School of Nursing and Health, Zhengzhou University, Zhengzhou, Henan, China; ^2^ College of Public Health, Zhengzhou University, Zhengzhou, Henan, China; ^3^ Department of Neurology, the First Affiliated Hospital of Zhengzhou University, Zhengzhou, Henan, China

**Keywords:** anxiety, depression, sleep problems, network analysis, health-promoting lifestyles, university students

## Abstract

**Background:**

Anxiety, depression, and sleep problems are prevalent comorbid mental disorders among university students. The World Health Organization (WHO) emphasized a mental health promotion objective, recommending the consideration of protective health-promoting factors in strategies aimed at preventing mental disorders. Integrating theoretically significant constructs (such as protective factors) enhances our comprehension of the intricate mechanisms that underpin mental disorders. This study employed network analysis to first identify core and bridge symptoms within comorbid mental disorders and then explore how health-promoting lifestyles (HPLs) were associated with these disorders. The ultimate goal is to offer health promotion recommendations to enhance students’ quality of life.

**Methods:**

A total of 3,896 qualified university students participated in this study. Anxiety, depression, sleep problems, and HPLs were assessed using the GAD-7, PHQ-9, PSQI, and HPLP-II scales. A Gaussian Graphical Model was used to construct the networks. The *Network Comparison Test* was applied to determine whether the associations between HPLs and comorbid symptoms vary by gender, educational level, family sibling, and mental health status.

**Results:**

Low energy (PHQ4) had the highest strength centrality, followed by Daytime dysfunction (PSQI7) and Trouble relaxing (GAD4). Five bridge symptoms were identified: Daytime dysfunction (PSQI7), Self-harm even suicide (PHQ9), Sad mood (PHQ2), Low energy (PHQ4), and Feeling afraid (GAD7). Regarding protective HPLs, Physical activity, Spiritual growth, and Stress management generally emerged as the top three central mental health-promoting behaviors.

**Conclusion:**

Targeting core and bridge symptoms with timely and appropriate interventions can alleviate anxiety, depression, and sleep problems in this population. Moreover, promoting physical activity, fostering spiritual growth, and managing stress are likely to significantly enhance the overall mental health of university students.

## 1 Introduction

The mental health of university students has garnered growing attention. Faced with multifaceted pressures such as academics, social interactions, and career choices, more university students are taking leaves of absence or dropping out due to mental disorders (1). The Global Burden of Disease (GBD) Study highlighted the anxiety and depression have emerged as significant mental health challenges on a global scale (2). According to a systematic review conducted in 2022, the prevalence of anxiety and depression among university students has escalated to 39.0% and 33.6%, respectively (3). Importantly, sleep problems often occur concurrently with anxiety and depression. Since the revision of DSM-III-R by the American Psychiatric Association (APA), sleep disorder has been delineated as a distinct and specific category within DSM-IV, DSM-IV-TR, and DSM-5 (4). A meta-analysis among Chinese university students found a 25.7% prevalence of sleep disorders, with 20.3% dissatisfied with sleep quality and 23.6% exhibiting insomnia (5). During episodes of depression, university students suffer from diminished sleep quality, longer sleep onset latency, and increased daytime sleepiness (6). Furthermore, sleep problems and anxiety may be mediated by a pathogenic mechanism involving hyperarousal, influenced by imbalances in neurotransmitters such as cholinergic and gamma-aminobutyric acid (GABA) (7). In short, mental disorders typically co-occur in individuals rather than manifest independently, with those reporting depression and anxiety simultaneously exhibiting more sleep disturbances (8), a phenomenon referred to as comorbidity in clinical research.

Mental disorders also constitute a risk factor for self-harm and even suicide. Data from the International College Student Health Survey showed that approximately one-third of full-time student respondents have experienced at least one DSM-IV mental disorder (9). Compared to the general adult population, university students exhibit significantly higher rates of suicidal ideation (24% vs 9%) and suicide attempts (9% vs 2.7%) (10). Studies have proved that university students face greater challenges in mental health, highlighting the importance of conducting in-depth research on disorders such as anxiety, depression, and sleep. However, previous research on university students’ mental health has primarily relied on total scores from screening scales, often neglecting individual symptoms and the interactions between these symptoms. The network theory of mental disorder (NTMD), as proposed by Borsboom, offers a new perspective to better understand this complexity (11). This theory posits that the persistence of mental disorders are contingent upon the causal relationships among their symptoms. Significant causal relationships may potentially lead to feedback loops among symptoms, perpetuating and exacerbating each other. Using network analysis methods (NAM) in NTMD, symptoms are constructed into an interconnected network where central symptoms are more likely to trigger other symptoms, thereby playing a pivotal role in a disordered state of disease (12). Intervening on central symptoms may accelerate the deactivation of symptom interactions, thus improving peripheral symptoms, which holds considerable practical value (13). Current studies have widely applied this approach to investigate the network structure of mental symptoms among various populations, including the elderly, chronic disease patients, and healthcare workers (14–16).

From a universal health perspective, the World Health Organization (WHO) has proposed a mental health promotion objective, emphasizing that supportive social environment and healthy daily habits can help reduce the incidence of mental illnesses. Moreover, among five primary determinants of human health, lifestyle and behavioral factors contribute 60% (17). Health-promoting lifestyles (HPLs) refer to the multi-dimensional, spontaneous, and sustained daily activities undertaken with the aim of enhancing or promoting individual health and well-being (18). In clinical treatment, promoting the adoption of this adaptive health-promoting lifestyle is also considered a highly cost-effective and potentially applicable non-pharmacological intervention that may alleviate stress and depression (19). It showed that increasing high-intensity physical activity can alleviate the severity of mental health issues or reduce their occurrence among student populations (20). As a factor that promotes better sleep quality, cultivating healthy lifestyle habits is also an effective strategy for improving sleep (21). Certain lifestyles that emphasize mental growth, stress management, and interpersonal relationships are also negatively correlated with anxiety and depression (22). However, the prevalence of healthy behaviors among university students is currently suboptimal, with a considerable number engaging in health-risk behaviors and adopting unhealthy lifestyles (23). Hence, a comprehensive investigation into the correlation between depression, anxiety, sleep problems, and their protective factors (health-promoting lifestyles) among university students is necessary to better enhance their overall health.

Recently, researchers have also suggested moving beyond symptom measurement within networks. They propose including not only psychiatric symptoms but also a wider range of theoretically relevant components (such as risk or protective factors), to further understand the complex mechanisms promoting or preventing the development of mental disorders (24, 25). Risk factors provide valuable insights into understanding mental illness, yet the importance of protective factors is equally significant. For example, in a sample of 85 remitted depressed patients, resilience was shown to be an important protective factor, predicting fewer residual depression symptoms and less ruminative thinking (26). Eduardo and colleagues examined the relationship between adolescent suicidal behavior and socio-emotional indicators during adolescence, revealing the potential effectiveness of self-esteem and personal well-being in reducing suicide risk (27). By integrating potential health-promoting lifestyle choices with anxiety, depression, and sleep problems, the network model provides a more comprehensive approach to investigate the protective factors operating at the crossroads between health promotion and the development of mental disorders. Furthermore, it’s worthwhile to consider the variances concerning gender, educational level, familial sibling, and mental health status. Currently, multi-group network analysis has been developed to use the *Network Comparison Test* (NCT) based on resampling permutation tests to compare global strengths and edge connections between subgroups (28).

Recognizing the complexity of mental health challenges, our first aim is to (a) identify the symptoms that play significant roles in activating, maintaining, and connecting anxiety, depression, and sleep problems within the psychopathology network. Considering the continuously evolution of psychiatric research, our second aim is to (b) explore how comorbid symptoms and protective HPLs are interconnected, discover the central mental health-promoting behaviors, and investigate whether these associations vary by gender, educational level, familial sibling, and mental health status. Ultimately, this may better help educators and healthcare professionals develop evidence-based, more precise health promotion and non-pharmacological intervention strategies to address the mental health problems of university students.

## 2 Methods

### 2.1 Study settings and participants

This cross-sectional study, conducted from January 13 to March 18, 2022, was supported by the Psychological Health Education Center at Zhengzhou University. In China, the surveyed location was a Ministry of Education-designated world-class university and a first-class discipline construction university, consistently ranking among the top five nationwide in student enrollment each year, thereby ensuring the representativeness of the survey sample. We employed a multistage sampling method, using seven academic categories (e.g., Medicine, Science, Engineering) as primary units, and degree types (undergraduate and graduate) as secondary units, with stratified sampling throughout. Subsequently, classes served as third-stage units, with cluster sampling randomly selecting students from several classes within each combination of discipline and degree type. Similar to other studies (29, 30), the “Questionnaire Star” program integrated into WeChat was adopted to collect data. An electronic questionnaire was disseminated to 4,952 currently enrolled university students, resulting in the collection and inclusion of 4,698 responses, yielding a response rate of 94.87%. The questionnaire was distributed via the WeChat and Tencent QQ platforms. To ensure data quality, measures implemented: restricting each IP address to a single submission, excluding responses with completion times shorter than 240 seconds or longer than 10 minutes, eliminating incomplete questionnaires, and discarding those with contradictory answers. As a result, a total of 3,896 eligible participants were included. Prior to participation, all subjects provided electronic informed consent. This study received approval from the Ethics Committee of Zhengzhou University (ZZUIRB2022–06).

Power analysis was commonly employed to ascertain the requisite sample size for a study. According to Epskamp and Fried’s research (31), we utilized the *netSimulator* function to simulate data within a given network model and anticipated network structure, thereby exploring the appropriate sample size necessary for detecting genuine effect sizes. As the network encompasses a greater number of nodes, a larger sample size is necessitated to achieve an equivalent level of reproducibility. Consequently, we conducted data simulations for a network model encompassing comorbid symptoms and health-promoting lifestyles, with the result shown in **Supplementary Figure S1**. And when N = 1600, the network’s correlation and strength were above 0.9, and the effects on other metrics were also acceptable (above 0.7).

### 2.2 Measurements

#### 2.2.1 Generalized anxiety disorder scale-7

Using the 7-item Generalized Anxiety Disorder Scale (GAD-7) to measure the severity of anxiety symptoms (32). This scale includes seven items that correspond to the DSM-IV criteria for generalized anxiety disorder, with each item rated on a scale from 0 (not at all) to 3 (nearly every day). The total score ranges from 0 to 21, with higher scores representing more severe anxiety symptoms, while a score of 5 or above is classified as mild anxiety. The Chinese adaptation of the GAD-7 has undergone thorough validation, establishing its efficacy as a screening tool for anxiety within the general Chinese population (33).

#### 2.2.2 Patient health questionnaire-9

Depression symptoms were evaluated using the Patient Health Questionnaire-9 (PHQ-9) (34), which is based on the nine Diagnostic and Statistical Manual of Mental Disorders-IV (DSM-IV) criteria for major depression disorder (35). Participants responded to the items on a 4-point Likert scale ranging from 0 (not at all) to 3 (most of the time or always), resulting in a total score ranging from 0 to 27 across the nine items. For PHQ-9 scores, higher total scores represent more severe depression symptoms, with a score of 5 or above indicating mild depression. Extensively validated in the Chinese population (36), the PHQ-9 serves as an effective tool for assessing depression symptoms. Most previous studies have focused on sleep duration and insomnia symptoms (e.g., difficulty falling asleep or staying asleep), while paying little attention to sleep dimensions (e.g., sleep efficiency, daytime dysfunction, and sleep medication) (37). Therefore, we excluded the third item from this scale and employed the Pittsburgh Sleep Quality Index (PSQI) to comprehensively understand the complexity of sleep problems.

#### 2.2.3 Pittsburgh sleep quality index

The Pittsburgh Sleep Quality Index (PSQI) was used to assess the severity of sleep problems (38). Comprising 19 self-assessment items, the PSQI includes seven dimensions: (1) “Subjective Sleep Quality”; (2) “Sleep Latency”; (3) “Sleep Duration”; (4) “Sleep Efficiency”; (5) “Sleep Disturbance”; (6) “Use of Sleep Medication”; and (7) “Daytime Dysfunction”. In the Chinese population, a global PSQI score exceeding 7 indicates of poor sleep quality, with a sensitivity of 98.3% and specificity of 90.2% (39).

#### 2.2.4 Health-promoting lifestyle profile-II

The Health-Promoting Lifestyle Profile (HPLP) was developed to quantify the frequency of participation in health-promoting behaviors (40). The HPLP-II was a revision of the HPLP, consisting of a total of 52 items across six dimensions: interpersonal relationship, health responsibility, stress management, nutrition, physical activity, and spiritual growth. It employed a Likert 4-point scoring system, with higher total scores indicating better health behaviors. The Chinese version of the HPLP-II was revised to include 40 items and has been effectively validated in the Chinese population (41). This version was employed in our survey.

### 2.3 Statistical analysis

All statistical analyses were performed using the R-Studio program (version 4.3.2). First, descriptive statistical methods were used to summarize demographic information and item scores from each scale. Then, we constructed a comorbid network of anxiety, depression, and sleep problems, in addition to a comprehensive network of comorbid symptoms and their association with health-promoting lifestyles. Network analysis can establish bivariate relationship among multiple variables and reveal the structure and nature of entire complex system of mental disorders caused by the causal interactions among variables (including core components, co-occurrence relationships, and critical nodes), which cannot be explained by regression analysis and latent variable analysis (42).

Before constructing the networks, potential item redundancy was checked using the *goldbricker* function from the R package *networktools* (version 1.5.1). Following Jones’ manual (43), if the proportion of significantly different central correlations between two variables and other items is less than 25%, then it can be confirmed that these two items measure the same trait or symptom (i.e., redundancy).

#### 2.3.1 Network estimation

The Pairwise Markov Random Field (PMRF) is commonly employed in cross-sectional studies. Given the continuous nature of our dataset, we utilized the Gaussian Graphical Model (GGM) to estimate a network of partial correlation coefficients (44). The partial correlation network is a type of model based on weighted correlation networks. Partial correlation coefficient denotes the correlation between two nodes while holding all other information in the network constant, hence also referred to as “conditional independence correlations”. To mitigate spurious connections and enhance the network’s comprehensibility, we applied the graphical least absolute shrinkage and selection operator (LASSO) for regularization (45). The Extended Bayesian Information Criterion (EBIC) was used to identify the optimal model fit with a default tuning parameter of 0.5 (46). Additionally, using the Fruchterman-Reingold algorithm to compute the optimal layout of the network, nodes with stronger correlations are positioned closer together, while nodes with weaker correlations are mutually exclusive. For both network estimation and visualization, we relied on the R packages *qgraph* (version 1.9.8) and *bootnet* (version 1.5.6) (44, 47). In graphs, each item is represented as a node, and the connections between nodes are termed edges. The thickness of the edges represents the strength of the association, while the color indicates the direction: green for positive correlations and red for negative correlations.

Quantifying the significance of each node within a network necessitates the computation of centrality metrics, which elucidate the probability that the activation of one node will exert an influence on others (48). Commonly employed measures of node centrality encompass betweenness, closeness, and strength. Nevertheless, prior studies have demonstrated that betweenness and closeness are unreliable for ascertaining node importance (44, 49). Consequently, this study employed strength as the centrality metric. Strength centrality evaluates the aggregate absolute edge weights between a node and all its directly connected nodes, thereby describing the robustness of direct connections between nodes in the absence of intermediary nodes. Hence, nodes with higher strength can be interpreted as more central. Furthermore, bridge centrality metrics (such as bridge strength, bridge closeness, and bridge betweenness) were also evaluated using the bridge function in the R package *networktools* (version 1.5.1) to identify specific nodes that act as conduits connecting different communities within the network (50). Bridge strength quantifies the total absolute edge weight between a community node (such as “physical activity” in health-promoting lifestyles) and all other nodes outside its community. Finally, the R package *mgm* (version 1.2–14) was employed to calculate predictability, which indicates the extent to which the variation of a given node in the network can be predicted by the variation of its connected nodes (51). In the network layout, the area within the surrounding cycle of each node represents its predictability value.

#### 2.3.2 Network stability and accuracy

We assessed network stability and accuracy using the bootstrap method in the R package *bootnet* (version 1.5.6), relying on the following three processes. To ensure the stability of strength and bridge strength, we initially implemented a case-dropping bootstrap procedure (44). This involved selectively discarding portions of data without inducing significant alterations in the existing network, thus affirming its stability. Stability was quantified by computing the Correlation Stability Coefficient (CS-C), indicating the maximum sample proportion that could be removed. Typically, a CS-C value of 0.25 or higher is preferred, with values exceeding 0.5 considered particularly desirable (52). Subsequently, to assess the accuracy of edge weights, we employed a non-parametric bootstrap method to derive confidence intervals (CIs) (53). A narrower confidence interval signifies a more reliable network estimation. Finally, to evaluate potential disparities between edges or nodes, we conducted bootstrapped paired differences in edge weights and centralities of different nodes (44).

#### 2.3.3 Network comparison

To explore potential variances in network characteristics related to comorbid symptoms and health-promoting lifestyles among university students based on gender, educational level, family sibling, and mental health status, we classified gender as “Male” and “Female”; education level as “Undergraduate” and “Graduate”; family sibling status as “Only-child” and “Non-only-child”; mental health status as “Non-mentally disordered” and “At least one mental disorder”. Then, we applied a permutation test with 1000 iterations using the R package *Network Comparison Test* (version 2.2.2) to determine if there are statistical differences in global strength (the absolute sum of all edge weights) as well as network structure (the distributions of edge weights) among subgroups (28). Subsequently, following the adjustment for multiple comparisons using the Bonferroni-Holm correction, the differences in strength at the level of individual edges between both networks were compared.

## 3 Results

### 3.1 Study sample characteristics

The demographic characteristics of the overall sample are presented in **Table 1**. Among the 3,896 participants included in this study, the mean age was 21.92 years (SD = 2.56), with 1,587 (40.7%) being male and 2,309 (59.3%) female. Of these, 1,824 (46.8%) were undergraduates, and 2,072 (53.2%) were graduate students. Altogether, 1,485 (38.1%) reported experiencing anxiety (GAD-7 total score ≥ 5), 1,745 (44.8%) reported experiencing depression (PHQ-9 score ≥ 5), and 629 (16.1%) reported experiencing sleep problems (PSQI total score ≥ 8). **Supplementary Table S1** displays the mean, standard deviation (SD), skewness, kurtosis, strength, bridge strength, and predictability of all items across the scales. In both the comorbid network of anxiety, depression, and sleep problems, and the comprehensive network of comorbid symptoms and health-promoting lifestyles, no item was found to be redundant with any other item (i.e., showing less than 25% significantly different correlations).

**Table 1:** Sample demographic characteristics (n = 3896).

Variables	Mean/N	SD/%
**Age (year)**	21.92	2.56
Gender		
Male	1587	40.7
Female	2309	59.3
BMI (kg/m^2^)		
Underweight (≦ 18.4)	544	14
Normal weight (18.5–23.9)	2582	66.3
Overweight (≧ 24)	770	19.7
Residence		
Urban	1889	48.49
Rural	2007	51.51
Academic discipline		
Medical	711	18.2
Non-Medical	3185	81.8
Education level		
Undergraduate Student	1824	46.8
Graduate Student	2072	53.2
Marital status		
Single and without a partner	2868	73.6
Single with a partner	916	23.5
Married	112	2.9
Family sibling status		
Only child	1007	25.8
Non-only child	2889	74.2
Anxiety (GAD-7)		
No anxiety (0–4)	2411	61.9
With anxiety (5–21)	1485	38.1
Depression (PHQ-9)		
No depression (0–4)	2151	55.2
With depression (5–27)	1745	44.8
Sleep problems (PSQI)		
normal sleep (0–7)	3267	83.9
Experiencing sleep problems (8–21)	629	16.1

### 3.2 Network of anxiety, depression and sleep problems

**Figure 1:**
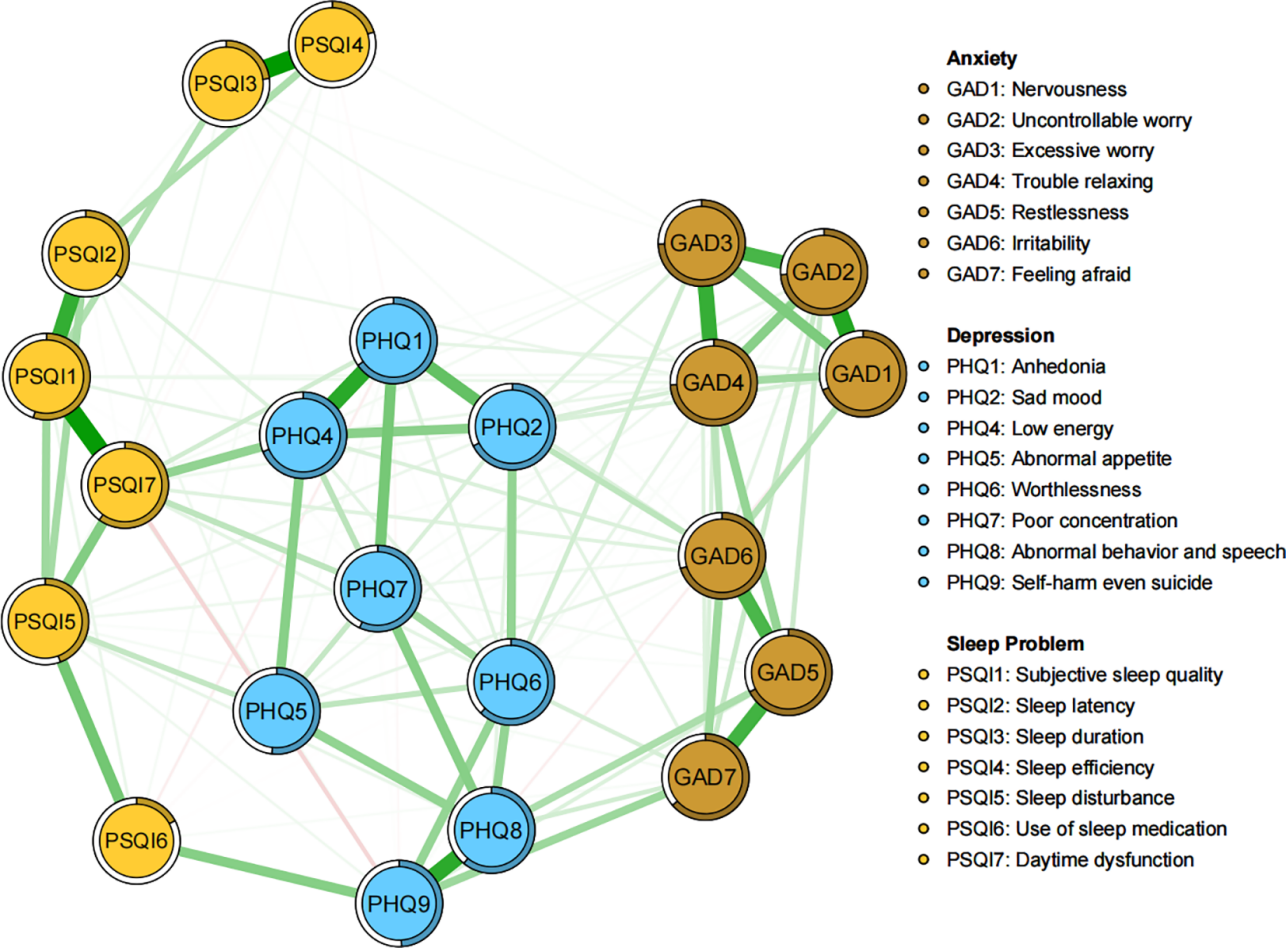
Network structure of anxiety, depression and sleep problems among university students.

As illustrated in **Figure 1**, within the network structure encompassing anxiety, depression, and sleep problems, there are a total of 131 non-zero edges out of 231 possible edges (network density = 0.57), with an average weight of 0.043. The five most prominent edges are all within specific communities: Sleep duration - Sleep efficiency (PSQI3 - PSQI4), Subjective sleep quality - Daytime dysfunction (PSQI1 - PSQI7), Nervousness - Uncontrollable worry (GAD1 - GAD2), Anhedonia - Low energy (PHQ1 - PHQ4), and Abnormal behavior & speech - Self-harm even suicide (PHQ8 - PHQ9) (**Supplementary Table S2**). Significant edges connecting different communities include Self-harm even suicide - Use of sleep medication (PHQ9 - PSQI6), Low energy - Daytime dysfunction (PHQ4 - PSQI7), and Feeling afraid - Self-harm even suicide (GAD7 - PHQ9) (**Supplementary Table S2**). Additionally, Excessive worry (GAD3) and Trouble relaxing (GAD4) exhibit the highest predictability (0.747, 0.742), whereas Use of sleep medication (PSQI6) has the lowest predictability (0.173). The average predictability is 0.56, indicating that, on average, more than half of the variance in the nodes can be explained by their adjacent nodes (**Supplementary Table S1**). Network stability and accuracy tests are shown in **Supplementary Figure S4**, with both strength and bridge strength centrality being 0.75, exceeding the recommended threshold of 0.5.

**Figure 2:**
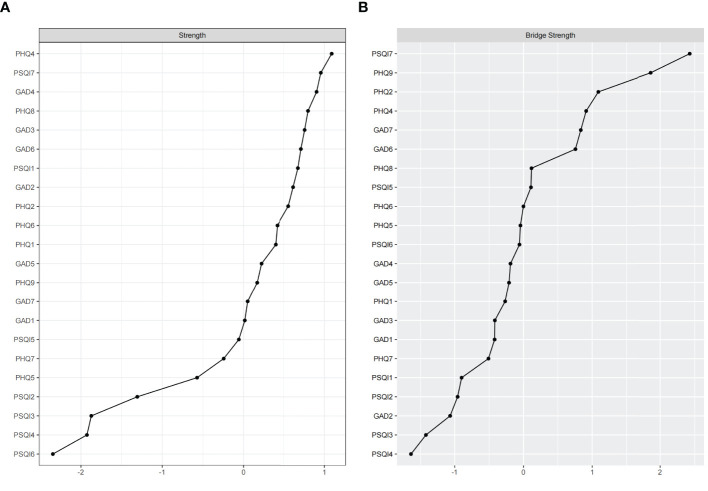
Standardized strength and bridge strength centrality of network structure of anxiety, depression and sleep problems among university students (z-scores). **(A)** strength centrality; **(B)** bridge strength centrality.

The centrality plot (**Figure 2A**) indicated that Low energy (PHQ4) exhibits the highest strength centrality, followed by Daytime dysfunction (PSQI7) and Trouble relaxing (GAD4), suggesting that these nodes occupy the most significant and influential positions within the comorbidity symptom network. According to Jones’ perspective (50), bridge symptoms are selected using the 80th percentile threshold for bridge centrality. As shown in **Figure 2B**, Daytime dysfunction (PSQI7), Self-harm even suicide (PHQ9), Sad mood (PHQ2), Low energy (PHQ4), and Feeling afraid (GAD7) exhibit the highest bridge strength, identifying them as the pivotal bridge symptoms linking anxiety, depression, and sleep problems comorbidity. The bootstrapped difference test (**Supplementary Figure S4**) for node strength and bridge strength further corroborate that these nodes are statistically significantly stronger than other nodes within the network.

### 3.3 Network of comorbid symptoms and health-promoting lifestyles

**Figure 3:**
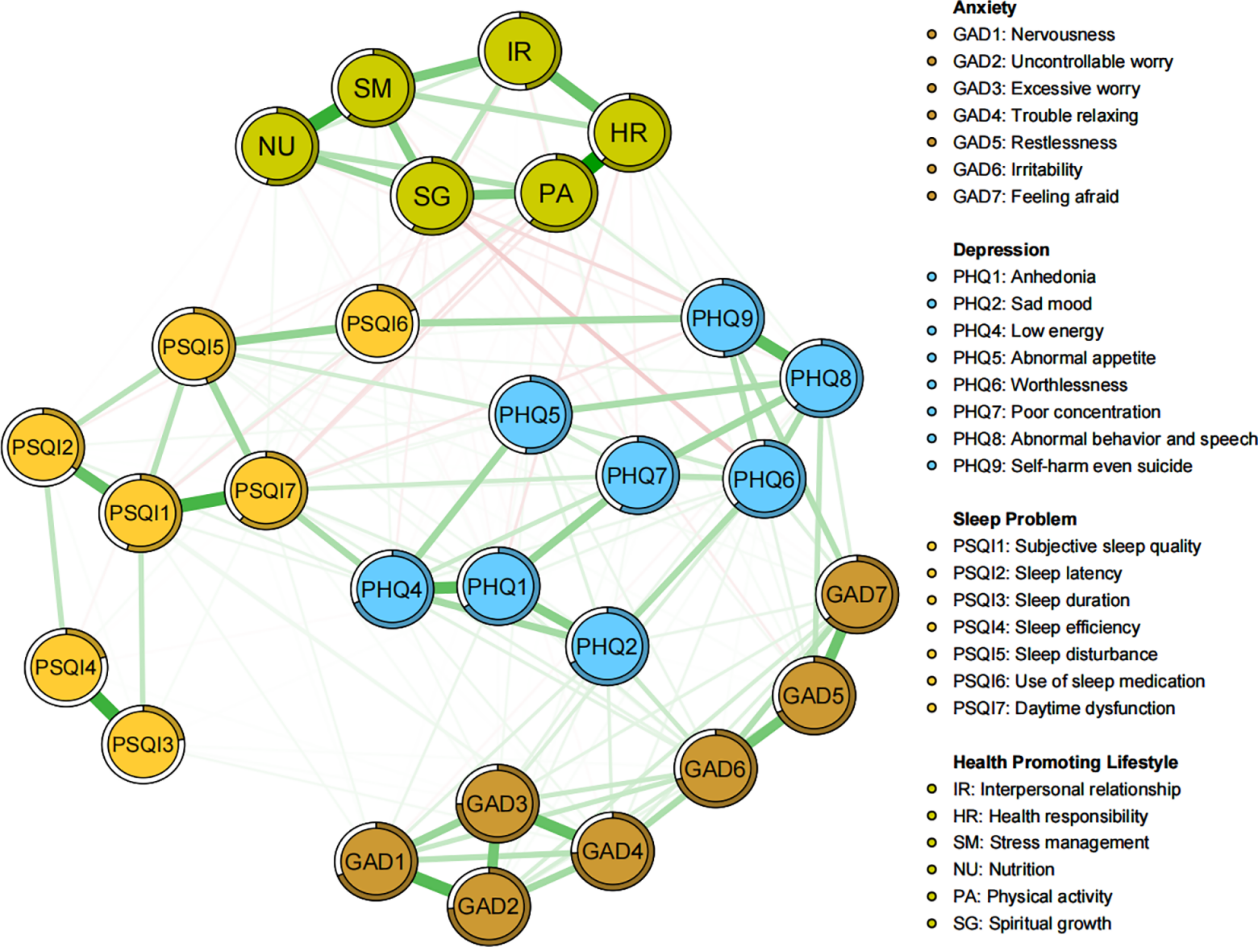
Network structure of comorbid symptoms and health-promoting lifestyles among university students.

The network of comorbid symptoms and health-promoting lifestyles is illustrated in **Figure 3**, with stability and accuracy tests presented in **Supplementary Figure S5**. Regarding strength centrality, Low energy (PHQ4), Daytime dysfunction (PSQI7), and Trouble relaxing (GAD4) remain the most significant and influential comorbid symptoms, while Physical activity, Health responsibility, and Stress management are identified as the top three central HPLs (**Supplementary Figure S2**). In terms of correlations, Spiritual growth and Physical activity exhibit the most negative edges with comorbid symptoms. Moreover, by evaluating the individual connection between each specific protective HPL node and the wider community of comorbid symptoms, Physical activity demonstrated the highest bridge strength. Specifically, Spiritual growth showed the strongest bridge strength with the depression community, Physical activity with the sleep problems community, and both Health responsibility and Stress management with the anxiety community (**Supplementary Table S3**; **Supplementary Figure S3**). However, as Health responsibility showed mostly positive correlations with anxiety symptoms. Thereby, Physical activity, Spiritual growth, and Stress management were identified as the central mental health-promoting behaviors.

### 3.4 Comparisons based on gender, education level, family sibling, and mental health status

NCT revealed the invariances of global strength and network structure among the four groups (**Supplementary Figures S6–S9**). In terms of global strength comparisons across the four subsamples, significant difference was only found in mental health status (non-mentally disordered: 8.72 vs at least one mental disorder: 13.21, *p* = 0.01). Other comparisons showed no significant differences (male: 13.19 vs female: 12.99, *p* = 0.634; undergraduate: 12.98 vs graduate: 13.43, *p* = 0.139; only-child: 13.23 vs non-only-child: 13.52, *p* = 0.733). Regarding network structure comparisons, significant differences were observed in familial sibling status (*M* = 0.16, *p* = 0.030) and mental health status (*M* = 0.28, *p* = 0.01); The other two subsamples showed no significant differences (gender: *M* = 0.13, *p* = 0.129; educational level: *M* = 0.12, *p* = 0.158). Additionally, after applying the Bonferroni-Holm correction, all edge weights in the three subsamples remained non-significant (*p* > 0.05).

## 4 Discussion

In the present study, we evaluated the core and bridge symptoms within comorbid network of anxiety, depression, and sleep problems, and identified central mental health-promoting behaviors. Consistent with epidemiological findings in university student populations, at least one in three individuals reported anxiety or depression, and one in every five to six individuals experienced sleep problems. Investigating the relationship between sleep problems and anxiety and depression from sleep dimensions (e.g., sleep efficiency, daytime dysfunction) provides a deep understanding of the complexity of mental health. Health promotion involves enabling individuals to achieve optimal physical and mental well-being, live under healthy habits, and make choices conducive to health (54). By applying a more comprehensive network approach, we can intricately analyze the associations between health-protective factors and specific mental disorders. In the following, we discuss the results of the networks.

### 4.1 Network results for anxiety, depression and sleep problems

Low energy (PHQ4) exhibited the highest strength centrality, indicating its crucial role in activating and sustaining the comorbid network of depression, anxiety, and sleep problems. This phenomenon was also widely observed among Chinese nurses and nursing students and closely associated with Anhedonia (PHQ1) (55, 56). Low energy, manifesting as fatigue or exhaustion, is a core feature of depression (57). During the late stages of the COVID-19 pandemic, the prevalence of fatigue among nursing students reached 67.3% (58). Evidence suggested that this may be associated with the somatization of psychological distress, particularly prevalent among Asian adolescents. This cultural tendency favors physical expression over verbal expression as a coping strategy (55). The cumulative impact of intense academic pressures, limited recreational and social activities, and inadequate sleep on their physical and mental health often manifested as a state of low energy. Ariel et al. found that low energy as a crucial predictor across multiple domains of health and functional impairment, corroborating our findings (59). It may also serve as an early indicator of anhedonia. Neurological evidence indicated that energy depletion may inhibit the neural systems responsible for enhancing the reception of rewarding stimuli (60). Furthermore, as the second most central symptom, Daytime dysfunction (PSQI7) showed strong edge connectivity with Low energy (PHQ4), serving as bridge symptoms. This supported findings from studies on Macau residents, migrant Filipino domestic workers, and clinicians with depression symptoms (61–63). It has been identified as a prominent bridge symptom linking childhood trauma, sleep disorders, and depression in adolescents (64). Exploratory research into sleep structure revealed that sleep problems could be categorized into nocturnal symptoms and daytime consequences, both contributing to dissatisfaction with sleep quantity or quality. However, interference with daytime functioning was centrally positioned and may be a critical outcome measure in insomnia treatment (65). Related studies indicated that students with low energy are more sensitive to stressors, amplifying the negative consequences of insomnia rather than the experience of insomnia itself (66, 67). The clinical significance of Trouble relaxing (GAD4) has been well-documented in insomnia and psychopathology (59). Notably, trouble relaxing was highly correlated with restlessness and clusters within the psychomotor community. Our results also showed that these symptoms have the highest predictability within the network, corroborating Bai et al.’s finding that difficulty relaxing ranks second in EI, with restlessness showing the highest BEI after sleep problems (63). The inability to relax as a core nexus symptom in comorbid PTSD, depression, and anxiety among trauma-exposed samples (68). Outside the classroom and internship settings, interventions such as mindfulness-based approaches and relaxation exercises can alleviate students’ physical and mental stress (69).

Node bridge strength centrality may provide insights for identifying critical bridge symptoms in the connection and progression of mental disorders. Beyond low energy and daytime dysfunction, Self-harm even suicide (PHQ9), Sad mood (PHQ2), and Feeling afraid (GAD7) also emerged as bridge symptoms within the current network. As a global health concern, Self-harm even suicide (PHQ9) has consistently been recognized as a key intervention target within adolescent mental disorders (70). Numerous scholars have probed into the nexus between adolescent mental health and suicidal ideation, revealing anxiety as a prospective predictor of suicidal ideation; over 50% of completed suicides and 20%-48% of suicide attempts are attributed to depression (71). We also identified that within the depression community, suicidal ideation is most strongly associated with abnormal behavior & speech (i.e., psychomotor agitation and impulsivity). Among adults experiencing major depression episodes, risk-taking behavior and psychomotor impulsivity were believed to facilitate the transition from suicidal ideation to suicide attempts (72). Our study also identified Feeling afraid (GAD7) as another significant bridge symptom, closely related to suicidal ideation. Epidemiological investigations signify that 75% of lifetime mental disorders manifest before the age of 24, with anxiety disorders typically emerging from early adolescence to young adulthood (73). The factors unique to college students, such as student debt, employment uncertainty, academic pressure, and separation from family, showed that feeling afraid doubles the likelihood of suicidal ideation among those experiencing anxiety symptoms (74). Additionally, Sad mood (PHQ2) played a distinct bridge role in linking anxiety disorders, as corroborated by a bayesian network of anxiety-depression bridges (75). Stigma toward mental illnesses could also lead to the emergence of adolescent emotional problems (76).

### 4.2 Network results for health-promoting lifestyles

The “Healthy China Action (2019–2030)” plan, formulated by the Healthy China Initiative Committee emphasizes the “big health” concept, integrating prevention and treatment through 15 major health promotion actions, shifting the focus from disease-centric to health-centric approaches. This offered valuable insights for our research. We found that physical activity occupied a central position in the network, potentially at the intersection of mental disorders and health. It remained the most prominent health node when quantifying the degree to which specific health-promoting behaviors correlate with changes in comorbid symptoms. Specifically, physical activity showed a significant negative correlation with sleep problems, potentially reducing the use of hypnotics, alleviating daytime dysfunction, and improving subjective sleep quality. Numerous scholars have corroborated our findings. It enhanced dopamine levels, altering responses to emotional events, and serving as a stress relief mechanism to help individuals return to pre-stress levels more quickly (77, 78). Moreover, adhering to scientific and effective physical exercise can regulate serum cortisol to optimal levels, boost immunity, and serum cortisol is closely linked to the body’s circadian rhythm (79). Studies also indicated that while body temperature decreases when falling asleep, evening exercise initially raises deep body temperature and accelerates the rate of temperature decline, thereby improving sleep (80). It’s noteworthy that whether a U-shaped relationship exists between physical activity and mental health (81), or the optimal frequency and intensity, requires further research to ascertain.

Spiritual growth can significantly reduce levels of depression. It’s strongly negatively correlated with self-harm even suicide, and worthlessness, marking these as the strongest negative edges within the network. Spiritual growth encompasses pursuing long-term life goals, experiencing oneself as positively developing and changing, gaining a sense of belonging, and maximizing the potential for a healthy life through goal-directed efforts (41, 82). Importantly, signs of depression are also characteristic of a lack of spirituality, Leung found that spiritual development significantly reduced depression, anxiety, and stress in adolescents while enhancing their self-confidence (83). It has been observed that young individuals experiencing depression often exhibit intrusive rumination, characterized by involuntary and repetitive contemplation of negative thoughts, which exacerbates their suffering (84). In contrast, spiritual growth can facilitate deliberate rumination, where individuals purposefully reflect on and analyze traumatic events to understand their meaning and impact, thereby promoting psychological growth (85). Additionally, self-efficacy is closely related to spiritual growth and can be viewed as an outward manifestation of it. It also serves as a mediator through which spiritual growth positively influences mental health, significantly reducing symptoms of depression, anxiety, and externalizing behaviors (86). As the third major health-promoting node, stress management showed a significant negative correlation with anxiety symptoms, alleviating trouble relaxing and restlessness. In this study, trouble relaxing was identified as a core symptom of anxiety, and restlessness was closely linked to it. This illustrates how stress management, by targeting core symptoms, can effectively and directly mitigate anxiety. Adolescents who frequently employ stress management strategies and adopt more positive coping and defense mechanisms exhibit fewer anxiety symptoms over time, underscoring the importance of incorporating stress management techniques into mental health programs (87).

The NCT analysis and quantification of centrality indices have confirmed the universal applicability of stress management, physical activity, and spiritual growth as key health-promoting factors for students. Nonetheless, some distinctions emerged when comparing the intensity levels of health-promoting behaviors’ effects on mental health symptoms across subgroups. Regarding gender, we found no association between stress management and anxiety in males, while physical activity significantly reduced anxiety and improved sleep quality. This implies that men often employ ineffective strategies for managing stress. Traditional masculine traits, which emphasize strength and control, condition men to suppress vulnerable emotions like fear and sadness, a habit persisting into adulthood (88). Additionally, a report found that men more frequently engage in regular exercise compared to women (89). For graduate students, stress management was the most central health-promoting behavior, surpassing physical activity. Among only-children, stress management strategies were more effective in alleviating anxiety compared to those with siblings. We hypothesize that for individuals experiencing greater and more complex stress, targeted stress management strategies are more direct and effective in reducing psychological issues in the short term. Our findings align with epidemiological surveys on students’ mental health (90–92). A higher proportion of women (41.14%) than men (33.71%) experienced anxiety. Anxiety severity was higher among graduate students (8.32) compared to undergraduates (8.09), and higher among only-children (8.75) compared to those with siblings (8.03). Notably, maintaining health responsibility (i.e., continuously attending to physical and mental health and remaining vigilant for potential illnesses) can counterintuitively exacerbate anxiety among women and graduate students. While this represents a form of health responsibility, over attention to health issues in high-stress populations can amplify anxiety, a phenomenon known as “health anxiety”. When classified by clinical scores, university students with at least one mental disorder exhibited significantly higher network density (0.44) compared to those without mental disorders (0.33). This elevated connectivity reflects the complexity and interconnection of mental disorders. Moreover, within the network of students experiencing mental disorders, stronger associations were observed among Abnormal behavior & speech - Self-harm even suicide, Spiritual growth - Worthlessness, and Stress management - Restlessness. Self-harm even suicide emerged as the second most central symptom node, following Low energy. Consistent with study on female nursing students (55), this finding suggested that psychomotor symptoms (such as psychomotor agitation/impulsivity and restlessness) and worthlessness may trigger connections within the network and significantly predict suicidal ideation. This also indirectly supports the notion that targeted interventions in stress management and spiritual growth are more directly effective under severe mental disorders.

## 5 Strengths and limitations

This study, based on Chinese university students, employed network analysis to identify core and bridge symptoms in three prevalent mental disorders. It uniquely integrated health-promoting behaviors into the network, exploring their associations with mental disorders, and identifying optimal nodes for health promotion, aligning with the WHO’s mental health promotion objective. The analysis revealed that central health-promoting behaviors predominantly impact the core and bridge symptoms of mental disorders, thereby preventing their development. Network comparison tests were conducted to further understand the differential effects or generalizability of these behaviors within the student population. Moreover, the HPLP-II questionnaire, chosen for its validated advantage in assessing self-maintenance health behaviors in daily life among the Chinese population, proved beneficial.

Despite these strengths, several limitations must be acknowledged. Firstly, the cross-sectional design of the study precludes causal inferences; longitudinal data are required to elucidate the complex mechanisms underlying the interactions between symptoms and health-promoting behaviors. Secondly, future research should incorporate risk factors to gain a comprehensive understanding, as risk factors may exacerbate mental disorders while protective factors might counteract these effects. Thirdly, our study was conducted in a single comprehensive university; despite efforts to mitigate bias, caution is warranted when generalizing the findings. Finally, it must be recognized that all sample information in this study was self-reported, which may affect the accuracy of the analysis.

## 6 Conclusion

In conclusion, university students are a high-risk group for mental disorders, with at least one in three reporting anxiety or depression, and one in every five to six experiencing sleep problems. Network analysis of the three common mental disorders revealed core symptoms: Low energy (PHQ4), Daytime dysfunction (PSQI7), and Trouble relaxing (GAD4). Bridge symptoms: Daytime dysfunction (PSQI7), Self-harm even suicide (PHQ9), Sad mood (PHQ2), Low energy (PHQ4), and Feeling afraid (GAD7). These symptoms are crucial in activating, maintaining, and interconnecting comorbid conditions. By incorporating health-promoting lifestyles (HPLs) into the network, we explored how protective HPLs are associated with these disorders and examined variations by gender, educational level, family sibling, and mental health status. Our findings highlighted the universal applicability of physical activity, spiritual growth, and stress management as the top health-promoting nodes for students. This study provided insights into common mental disorders and influential health lifestyles, which can aid educators and healthcare professionals in developing evidence-based, non-pharmacological intervention strategies to better promote mental health among university students.

## Data availability statement

The original contributions presented in the study are included in the article/**Supplementary Material**. Further inquiries can be directed to the corresponding author.

## Ethics statement

The studies involving humans were approved by The Ethics Committee of Zhengzhou University. The studies were conducted in accordance with the local legislation and institutional requirements. The participants provided their written informed consent to participate in this study.

## Author contributions

ZZ: Writing – review & editing, Writing – original draft, Visualization, Validation, Supervision, Software, Resources, Methodology, Investigation, Formal analysis. CS: Funding acquisition, Data curation, Conceptualization, Writing – review & editing, Visualization, Supervision, Resources, Project administration. PZ: Writing – review & editing, Visualization, Supervision, Software, Methodology, Data curation. LW: Writing – review & editing, Visualization, Validation, Supervision, Project administration, Funding acquisition. QZ: Writing – review & editing, Visualization, Supervision, Project administration, Investigation, Funding acquisition. YG: Writing – review & editing, Visualization, Supervision, Resources, Investigation. LG: Writing – review & editing, Visualization, Supervision, Resources, Project administration. YL: Writing – review & editing, Visualization, Supervision, Investigation. PW: Writing – review & editing, Visualization, Supervision, Funding acquisition. BH: Writing – review & editing, Validation, Supervision, Data curation. ML: Writing – review & editing, Validation, Supervision. JD: Writing – review & editing, Validation, Supervision. YW: Writing – review & editing, Validation, Supervision. ZW: Writing – review & editing, Validation, Supervision. YQ: Writing – review & editing, Data curation, Supervision, Project administration.
