# Supplementary material for: Exploring the interconnections of anxiety, depression, sleep problems and health-promoting lifestyles among Chinese university students: a comprehensive network approach
Source: Front Psychiatry. 2024 Jul 15;15:1402680. doi: 10.3389/fpsyt.2024.1402680 (PMC11284064; doi:10.3389/fpsyt.2024.1402680)
Supplement: Supplementary file 2 [file DataSheet_1.pdf]

Supplementary Material

Exploring the interconnections of anxiety, depression, sleep problems and health-promoting lifestyles among Chinese university students: A comprehensive network approach

Table S1. Basic information of scales and Descriptive item statistics

Table S2. Correlation matrix of Anxiety, Depression and Sleep problems

Table S3. Bridge Strength values between Health-promoting lifestyles to Symptoms of Anxiety, Depression, Sleep problems

Figure S1. Power analysis simulation results of Comorbid symptoms and Health-promoting lifestyles network

Figure S2. Standardized strength centrality plot of Comorbid symptoms and Health-promoting lifestyles network (z-scores)

Figure S3. Standardized bridge strength centrality plot between Health-promoting lifestyles to Symptoms of Anxiety, Depression, Sleep problems (z-scores)

Figure S4. Network stability and accuracy results of Anxiety, Depression and Sleep problems

Figure S5. Network stability and accuracy results of Comorbid symptoms and Health-promoting lifestyles

Figure S6. Comparison of network structures based on gender

Figure S7. Comparison of network structures based on education level

Figure S8. Comparison of network structures based on family sibling status

Figure S9. Comparison of network structures based on mental health status

Table S1. Basic information of scales and descriptive item statistics

Items context	M	SD	Skewness	Kurtosis	Strength	Bridge strength	Predictability
GAD1: Nervousness	0.71	0.738	1.075	1.349	0.927	0.173	0.689
GAD2: Uncontrollable worry	0.55	0.725	1.421	2.048	1.044	0.091	0.740
GAD3: Excessive worry	0.63	0.749	1.221	1.457	1.071	0.174	0.747
GAD4: Trouble relaxing	0.57	0.746	1.391	1.885	1.1	0.203	0.742
GAD5: Restlessness	0.4	0.643	1.713	3.104	0.967	0.2	0.675
GAD6: Irritability	0.55	0.715	1.367	1.944	1.062	0.323	0.703
GAD7: Feeling afraid	0.38	0.634	1.86	3.789	0.934	0.333	0.637
PHQ1: Anhedonia	0.76	0.774	0.938	0.667	1.001	0.193	0.653
PHQ2: Sad mood	0.59	0.690	1.158	1.509	1.032	0.365	0.677
PHQ4: Low energy	0.7	0.77	1.053	0.913	1.136	0.343	0.688
PHQ5: Abnormal appetite	0.55	0.736	1.388	1.796	0.813	0.221	0.518
PHQ6: Worthlessness	0.47	0.695	1.602	2.644	1.005	0.227	0.623
PHQ7: Poor concentration	0.62	0.772	1.221	1.136	0.877	0.162	0.569
PHQ8: Abnormal behavior and speech	0.32	0.61	2.165	5.109	1.079	0.464	0.610
PHQ9:Self-harm even suicide	0.18	0.486	3.201	11.94	0.957	0.112	0.491
PSQI1: Subjective sleep quality	0.77	0.747	0.719	0.107	1.055	0.105	0.550
PSQI2: Sleep latency	1.1	0.933	0.462	-0.688	0.67	0.14	0.346
PSQI3: Sleep duration	0.42	0.785	1.872	2.576	0.56	0.046	0.231
PSQI4: Sleep efficiency	0.39	0.695	1.782	2.572	0.549	0.019	0.204
PSQI5: Sleep disturbance	0.76	0.674	0.588	0.285	0.913	0.241	0.443
PSQI6: Use of sleep medication	0.07	0.355	5.686	35.879	0.468	0.219	0.173
PSQI7: Daytime dysfunction	0.93	0.88	0.728	-0.169	1.11	0.535	0.601
IR: Interpersonal relation	14.61	3.882	-0.375	-0.339	-	-	-
HR: Health responsibility	24.14	8.430	0.68	-0.1	-	-	-
SM: Stress management	14.42	3.481	-0.068	-0.428	-	-	-
NU: Nutrition	18.05	3.938	-0.328	-0.134	-	-	-
PA: Physical activity	18.3	6.236	0.545	-0.182	-	-	-
SG: Spiritual growth	14.74	3.815	-0.311	-0.619	-	-	-

Table S2. Correlation matrix of Anxiety, Depression and Sleep problems

	GAD1	GAD2	GAD3	GAD4	GAD5	GAD6	GAD7	PHQ1	PHQ2	PHQ4	PHQ5	PHQ6	PHQ7	PHQ8	PHQ9	PSQI1	PSQI2	PSQI3	PSQI4	PSQI5	PSQI6	PSQI7
GAD1	0.000																					
GAD2	0.323	0.000																				
GAD3	0.190	0.266	0.000																			
GAD4	0.134	0.172	0.294	0.000																		
GAD5	0.000	0.075	0.000	0.154	0.000																	
GAD6	0.107	0.039	0.099	0.088	0.262	0.000																
GAD7	0.000	0.077	0.049	0.056	0.276	0.144	0.000															
PHQ1	0.037	0.000	0.008	0.000	0.000	0.052	0.000	0.000														
PHQ2	0.056	0.032	0.046	0.057	0.002	0.096	0.039	0.226	0.000													
PHQ4	0.000	0.000	0.020	0.029	0.000	0.051	0.000	0.313	0.171	0.000												
PHQ5	0.004	0.025	0.000	0.000	0.000	0.035	0.000	0.014	0.054	0.173	0.000											
PHQ6	0.000	0.012	0.067	0.035	0.000	0.010	0.057	0.044	0.156	0.039	0.093	0.000										
PHQ7	0.003	0.015	0.000	0.000	0.019	0.000	0.000	0.200	0.021	0.098	0.083	0.135	0.000									
PHQ8	-0.023	0.000	0.000	0.000	0.121	0.000	0.065	0.000	0.039	0.000	0.159	0.157	0.175	0.000								
PHQ9	0.000	0.007	-0.004	0.000	0.049	0.000	0.143	-0.012	0.000	0.000	0.017	0.154	-0.002	0.308	0.000							
PSQI1	0.020	0.000	0.000	0.027	0.000	0.000	0.000	0.000	0.000	0.037	0.024	0.003	0.000	0.000	0.000	0.000						
PSQI2	0.000	0.000	0.000	0.028	0.000	0.000	0.000	0.000	0.000	0.048	0.023	0.000	0.006	0.000	0.000	0.297	0.000					
PSQI3	0.000	0.000	0.007	0.024	0.000	0.010	0.000	0.000	0.000	0.000	0.000	0.005	-0.001	0.000	0.000	0.114	0.009	0.000				
PSQI4	0.000	0.000	0.008	0.000	0.000	0.000	0.000	-0.008	0.000	0.000	0.000	0.000	0.000	0.000	0.003	0.000	0.121	0.372	0.000			
PSQI5	0.007	0.000	0.000	0.000	0.008	0.024	0.016	0.000	0.028	0.000	0.087	0.000	0.017	0.032	0.022	0.140	0.131	0.000	0.016	0.000		
PSQI6	0.000	0.000	0.000	0.000	0.000	-0.001	0.012	-0.024	0.000	0.000	0.000	0.000	-0.006	0.000	0.177	0.028	0.000	0.000	0.015	0.205	0.000	
PSQI7	0.024	0.000	0.015	0.003	0.000	0.046	0.000	0.065	0.009	0.159	0.023	0.038	0.095	0.000	-0.059	0.364	0.006	0.018	-0.007	0.181	0.000	0.000

Table S3. Bridge Strength between Health-promoting lifestyles to Symptoms of Anxiety, Depression, Sleep problems

	Anxiety community	Depression community	Sleep problems community
Interpersonal relationship	0.000	0.038	0.047
Health responsibility	0.044	0.097	0.126
Stress management	0.041	0.039	0.012
Nutrition	0.005	0.038	0.074
Physical activity	0.016	0.106	0.148
Spiritual growth	0.000	0.207	0.035

Figure S1. The power analysis simulation results of Comorbid symptoms and Health-promoting lifestyles network

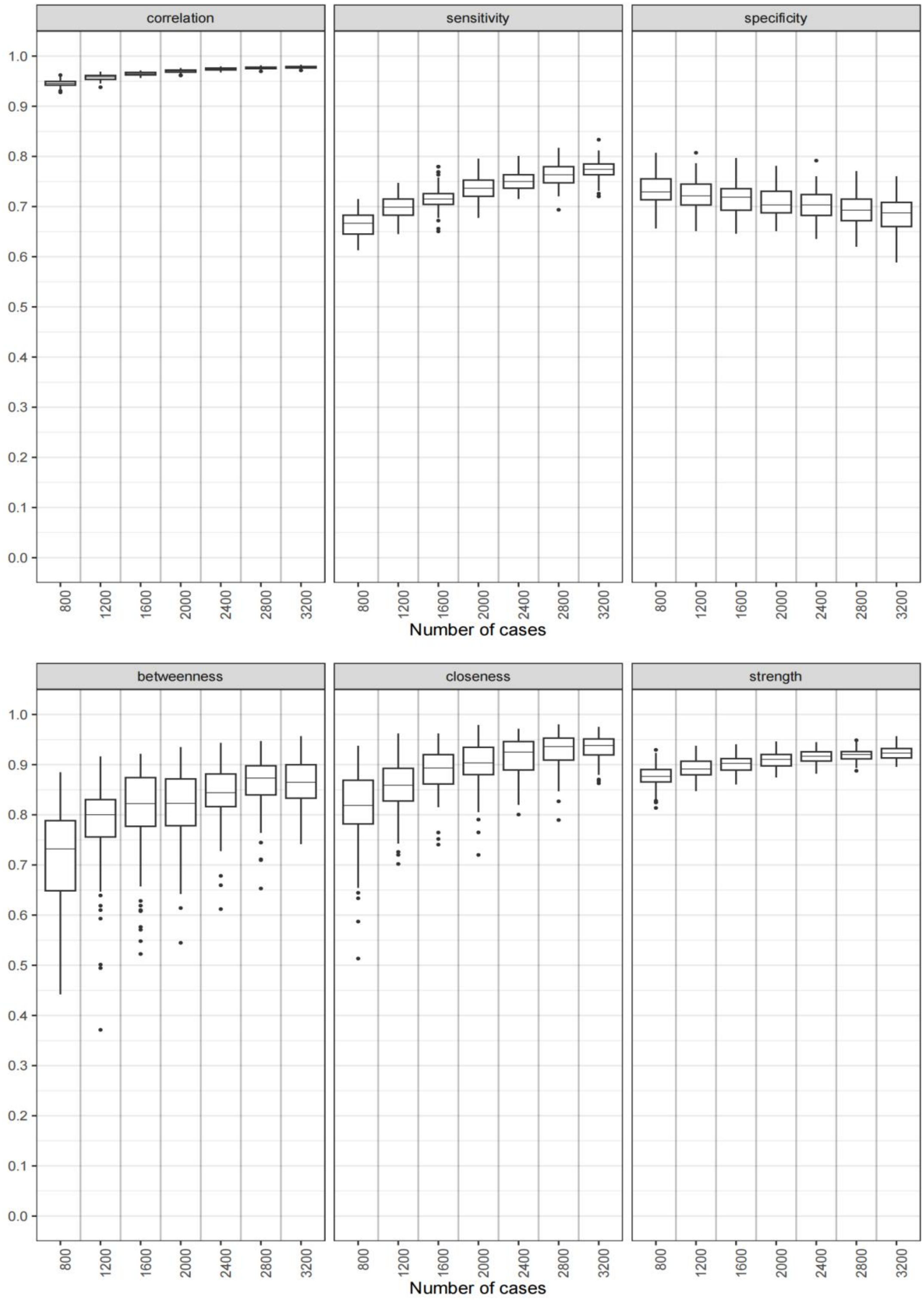

Figure S2. Standardized strength centrality plot of Comorbid symptoms and Health-promoting lifestyles network (z-scores)

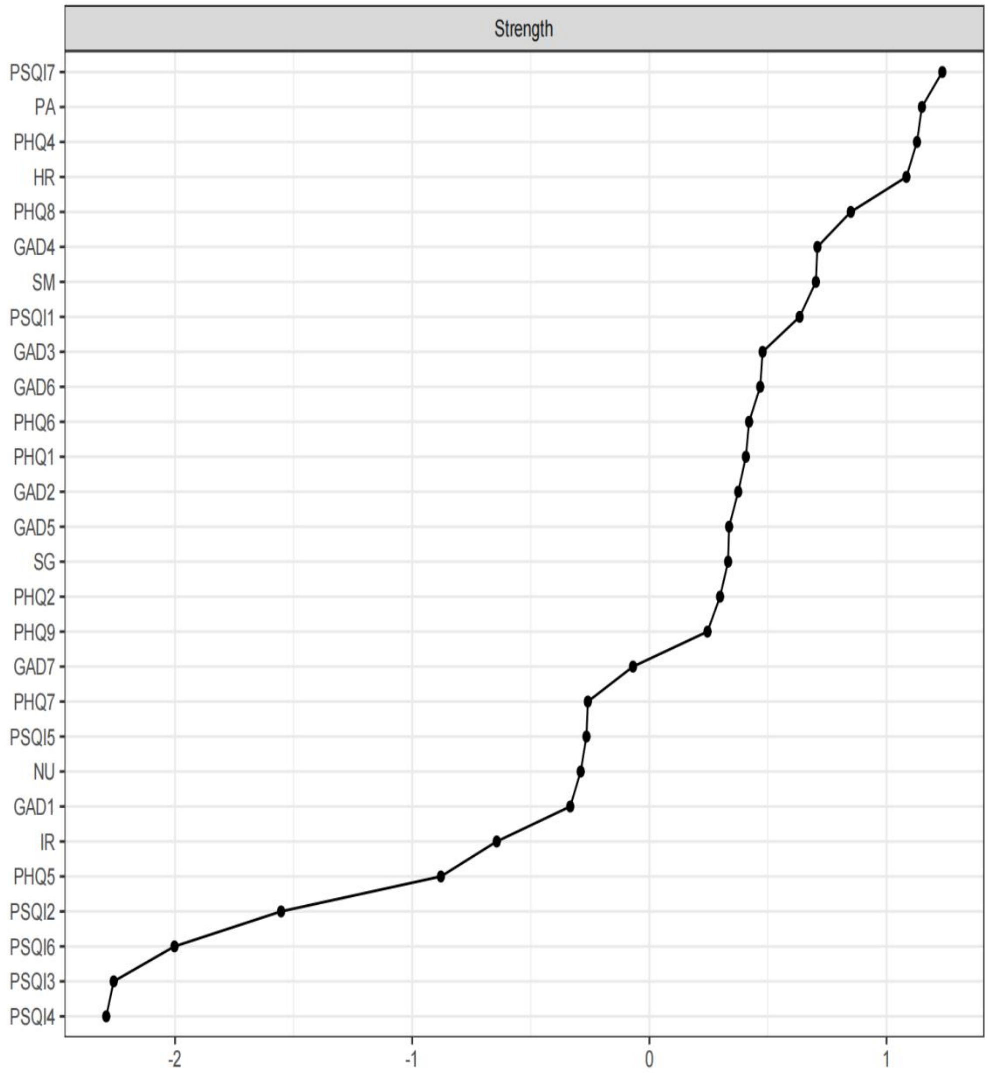

Figure S3. Standardized bridge strength centrality plot between Health-promoting lifestyles to Symptoms of Anxiety, Depression, Sleep problems (z-scores)

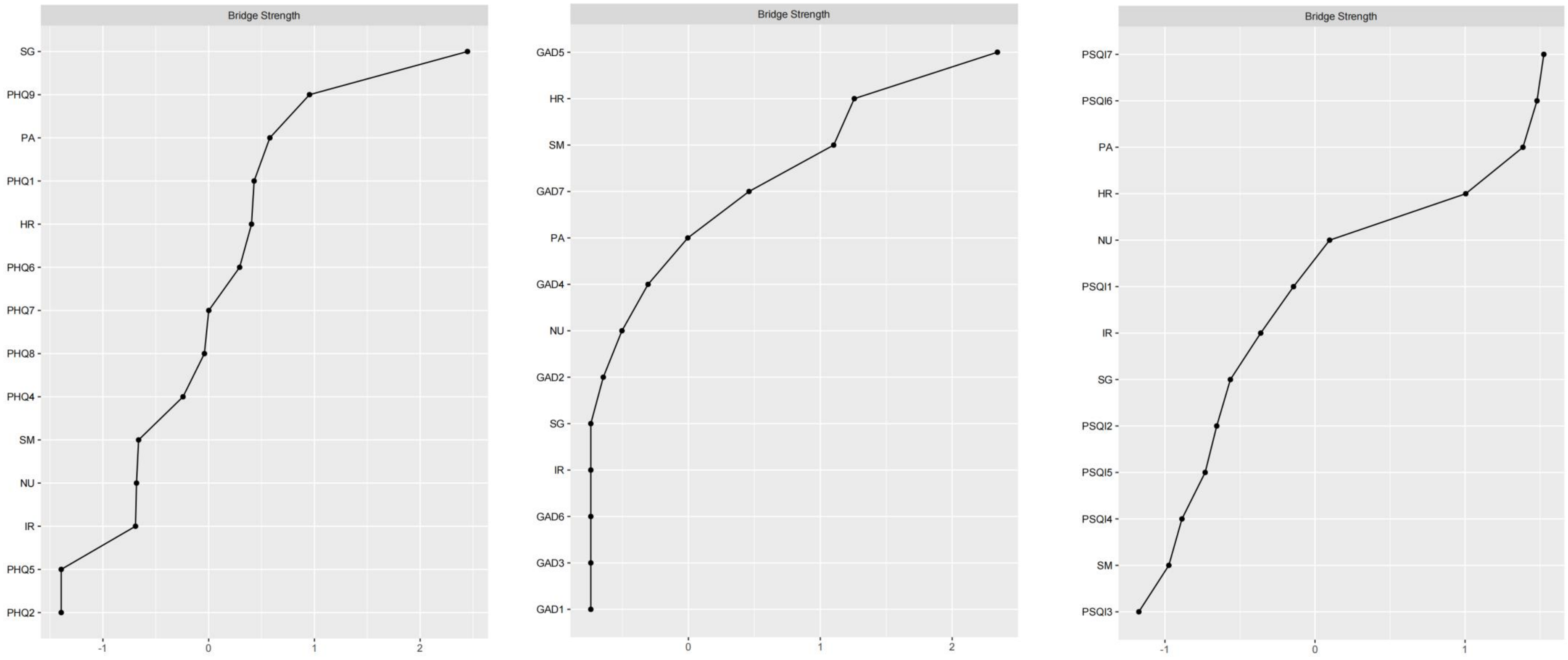

Figure S4. Network stability and accuracy results of Anxiety, Depression and Sleep problems

A:

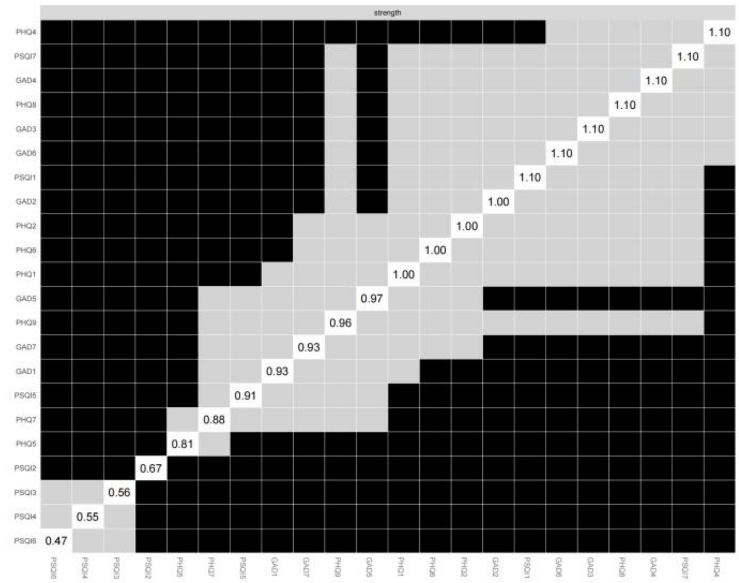

B:

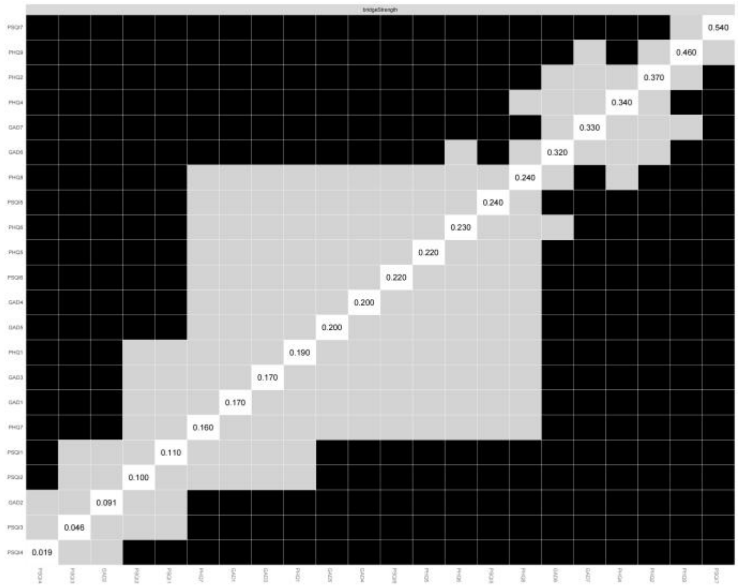

C:

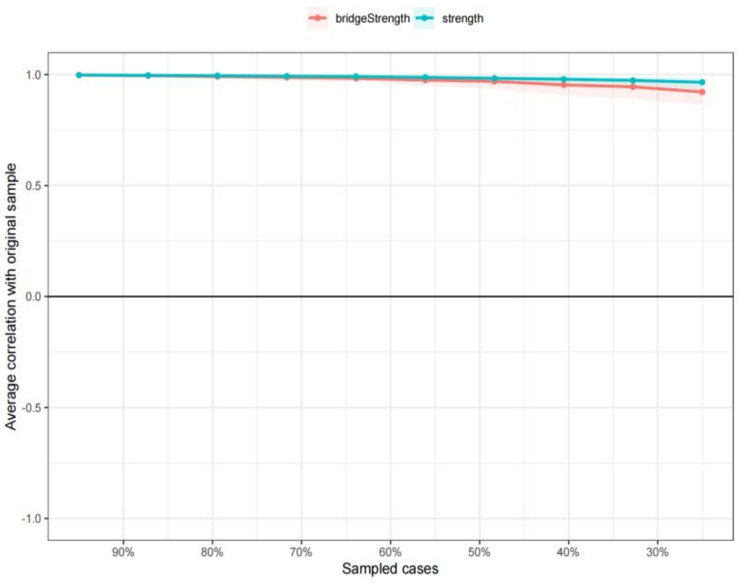

D:

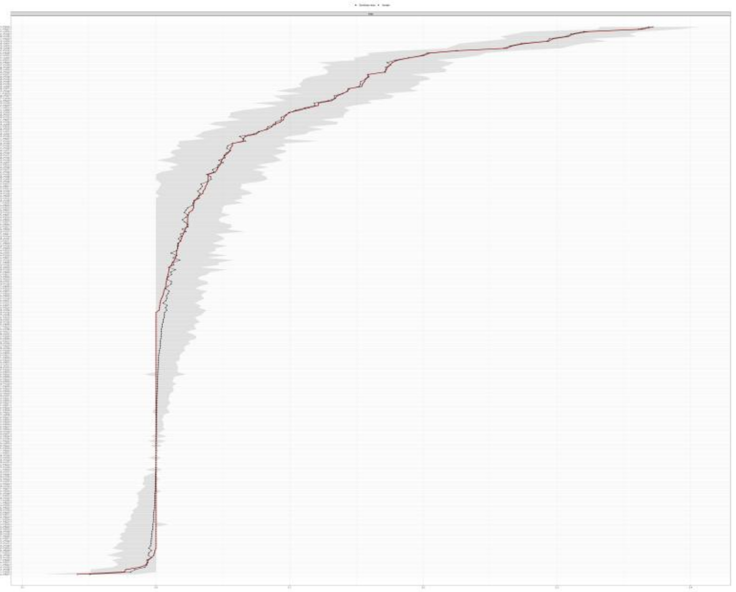

Note: (A) Nonparametric bootstrapped difference test for strength. (B) Nonparametric bootstrapped difference test for bridge strength. Gray boxes indicate node strength / bridge strength that do not differ significantly from one another, while black boxes indicate node strength / bridge strength that do differ significantly. The number in the white boxes (i.e., diagonal line) represent the value of node strength / bridge strength.

(C) Post-hoc stability analysis of node strength and bridge strength. The x-axis represents the percentage of cases of the original sample used at each step. The y-axis represents the average of correlations between the centrality metrics in the original network and the centrality metrics from the re-estimated networks after excluding increasing percentages of cases. The lines indicate the correlations of strength and bridge strength.

(D) Bootstrapped 95% confidence intervals of estimated edges. The red line represents the edge, as estimated in the sample. The grey indicates 95% bootstrapped confidence interval. The x-axis represents the edges, while specific edges are denoted along the y-axis by the grey lines.

Figure S5. Network stability and accuracy results of Comorbid symptoms and Health-promoting lifestyles

A:

B:

C:

D:

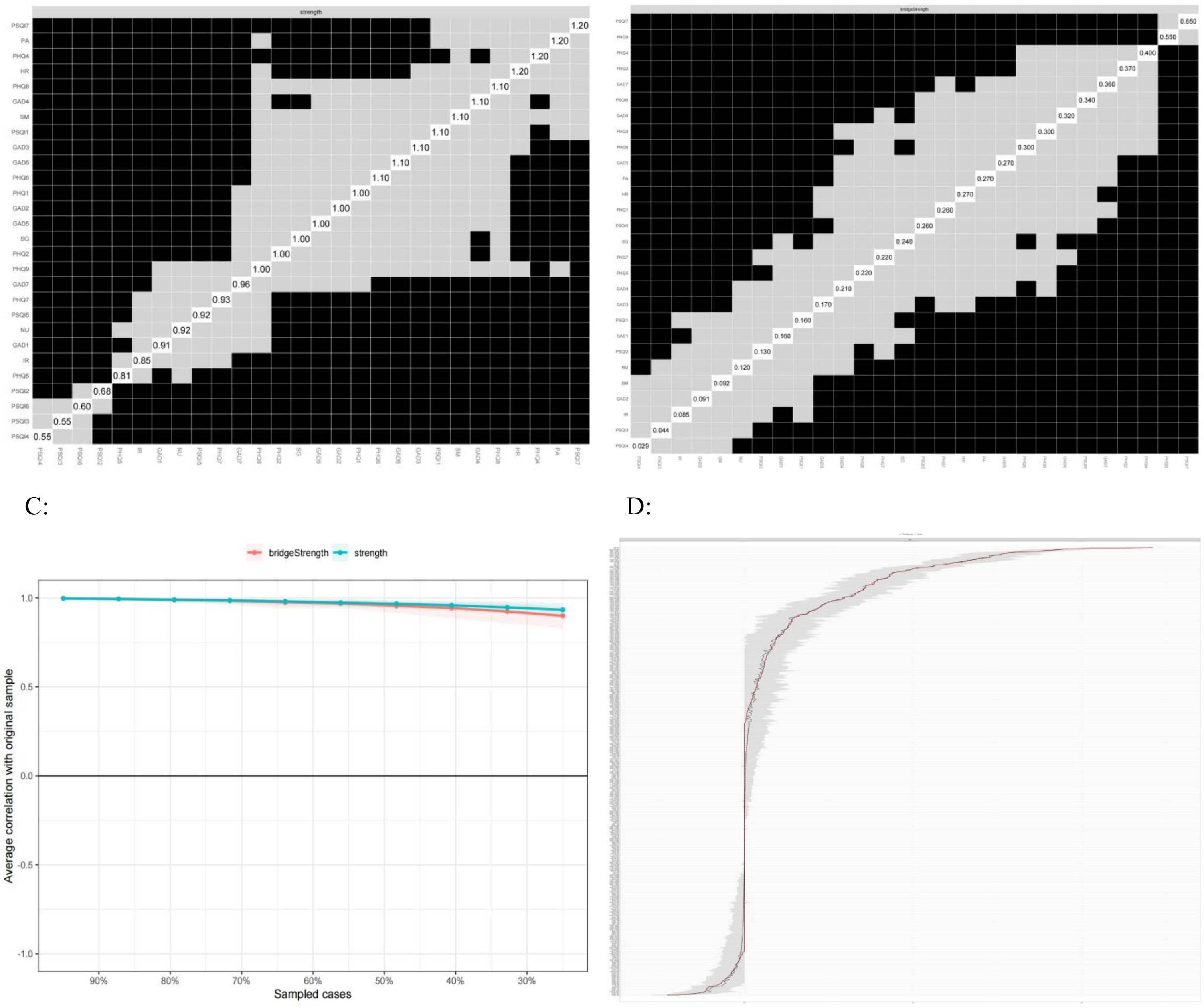

Note: (A) Nonparametric bootstrapped difference test for strength. (B) Nonparametric bootstrapped difference test for bridge strength. Gray boxes indicate node strength / bridge strength that do not differ significantly from one another, while black boxes indicate node strength / bridge strength that do differ significantly. The number in the white boxes (i.e., diagonal line) represent the value of node strength / bridge strength.

(C) Post-hoc stability analysis of node strength and bridge strength. The x-axis represents the percentage of cases of the original sample used at each step. The y-axis represents the average of correlations between the centrality metrics in the original network and the centrality metrics from the re-estimated networks after excluding increasing percentages of cases. The lines indicate the correlations of strength and bridge strength.

(D) Bootstrapped 95% confidence intervals of estimated edges. The red line represents the edge, as estimated in the sample. The grey indicates 95% bootstrapped confidence interval. The x-axis represents the edges, while specific edges are denoted along the y-axis by the grey lines.

Figure S6. Comparison of network structures based on gender

A:

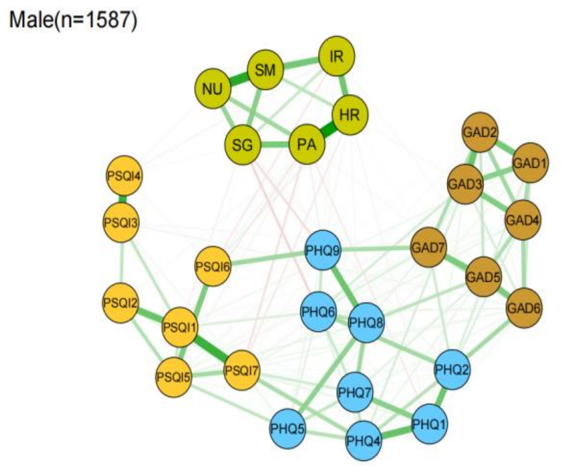

B:

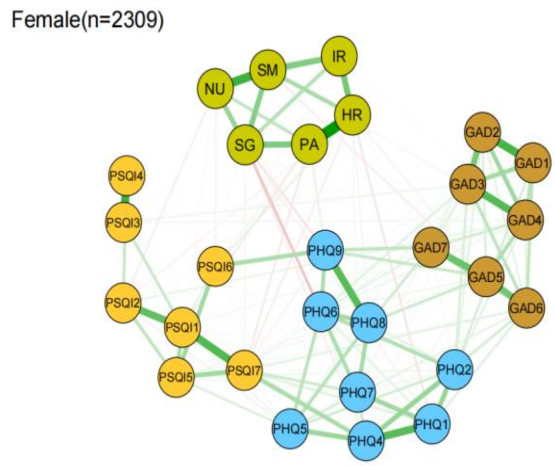

C:

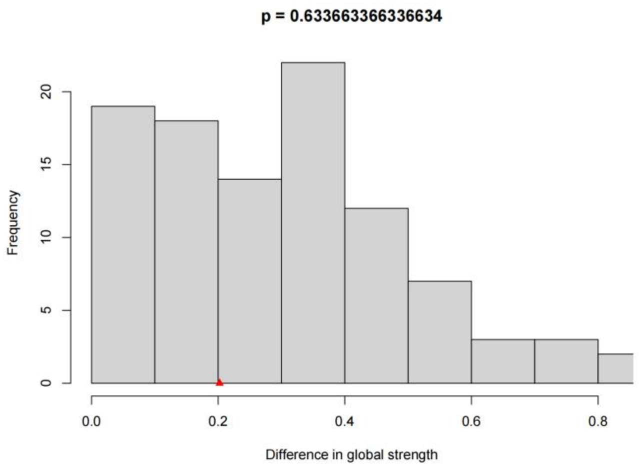

D:

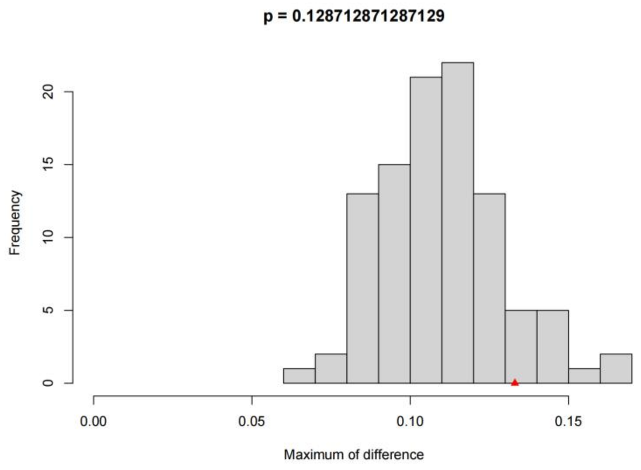

Note: (A) Estimated network model in males (n = 1587). (B) Estimated network model in females (n = 2309).

(C) A plot of bootstrap value of the difference in global strength. The difference was not significant (global strength among males: 13.19; among females: 12.99; $p = 0.634$).

(D) A plot of bootstrap value of the difference in network structure. The difference was not significant ($M = 0.13$, $p = 0.129$). Invariance in edge weights was tested using the permutation test, generating sets of p values for each edge-edge comparison. Bonferroni-Holm corrected p values were all > 0.05.

Figure S7. Comparison of network structures based on education level

A:

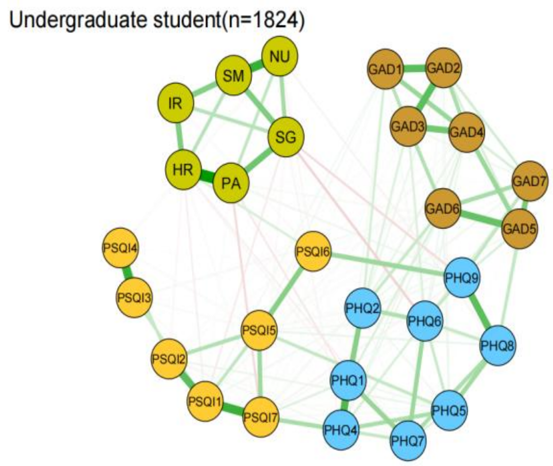

B:

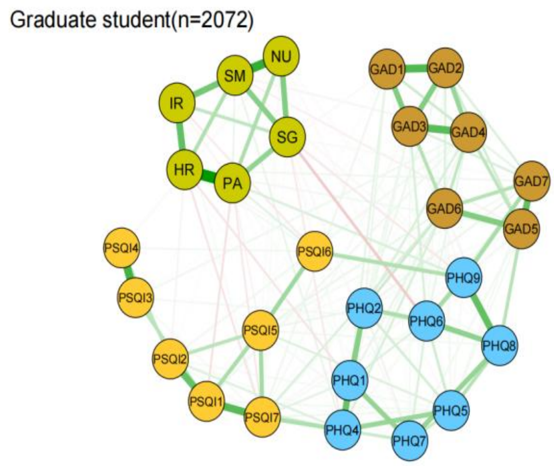

C:

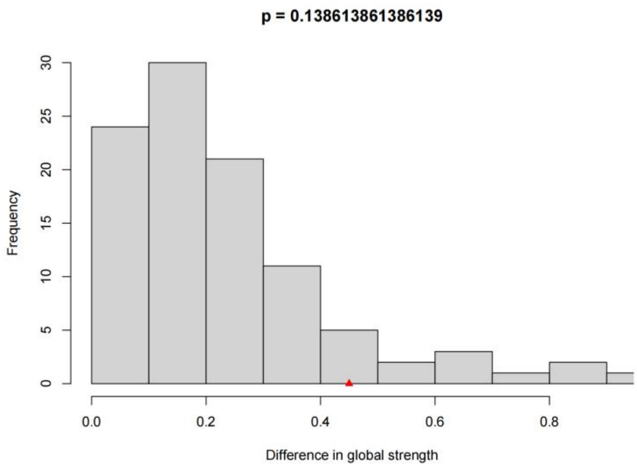

D:

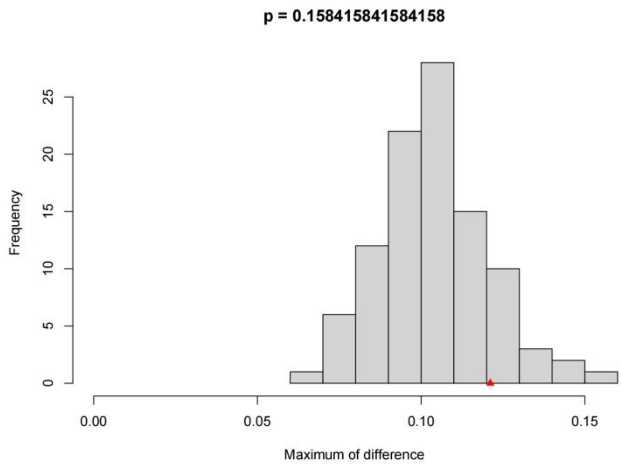

Note: (A) Estimated network model in undergraduate (n = 1824). (B) Estimated network model in graduate (n = 2072).

(C) A plot of bootstrap value of the difference in global strength. The difference was not significant (global strength among undergraduate: 12.98; among graduate: 13.43; $p = 0.139$).

(D) A plot of bootstrap value of the difference in network structure. The difference was not significant ($M = 0.12$, $p = 0.158$). Invariance in edge weights was tested using the permutation test, generating sets of p values for each edge-edge comparison. Bonferroni-Holm corrected p values were all > 0.05.

Figure S8. Comparison of network structures based on family sibling status

A:

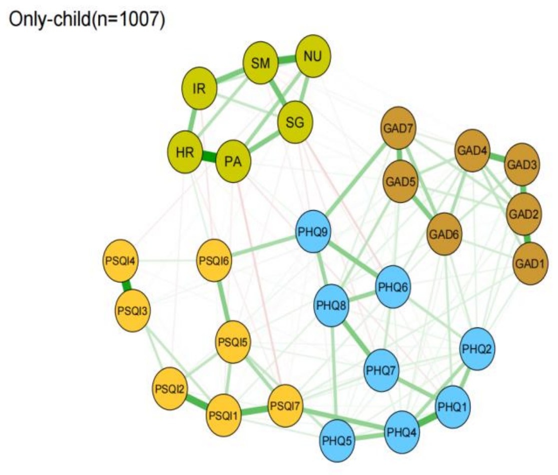

B:

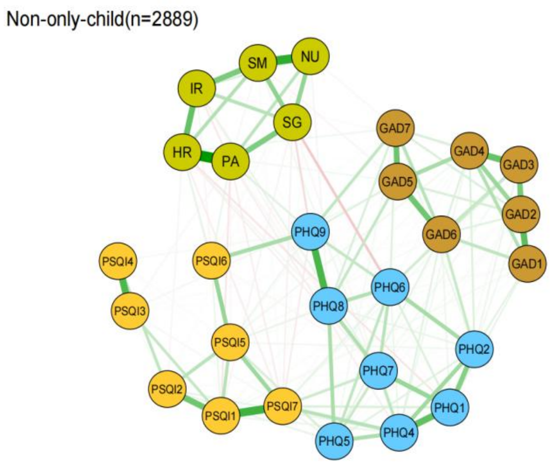

C:

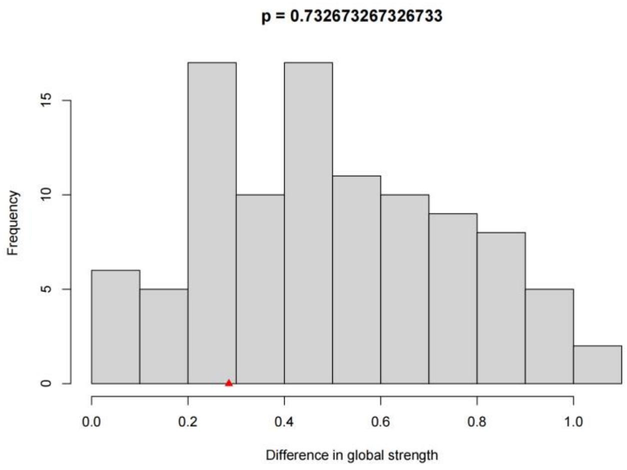

D:

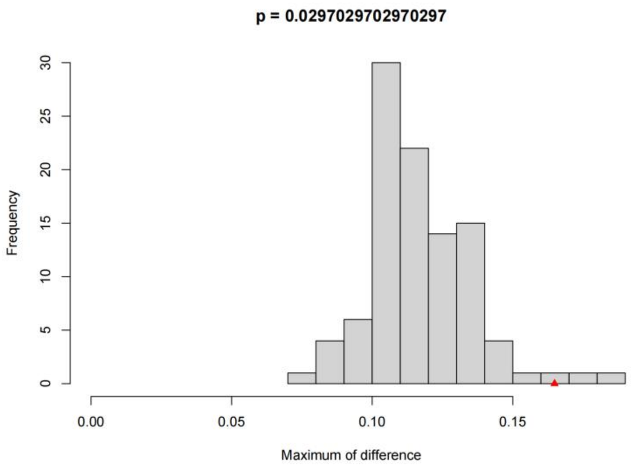

Note: (A) Estimated network model in only-child (n = 1007). (B) Estimated network model in non-only-child (n = 2889).

(C) A plot of bootstrap value of the difference in global strength. The difference was not significant (global strength among only-children: 13.23; among non-only-children: 13.52; $p = 0.733$).

(D) A plot of bootstrap value of the difference in network structure. The difference was significant ($M = 0.16$, $p = 0.030$). Invariance in edge weights was tested using the permutation test, generating sets of p values for each edge-edge comparison. Bonferroni-Holm corrected p values were all > 0.05.

Figure S9. Comparison of network structures based on mental health status

A:

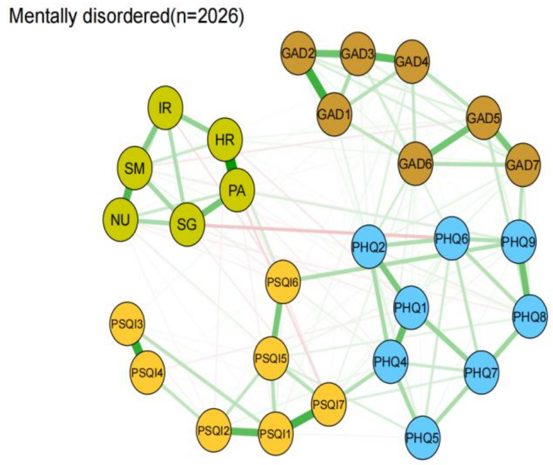

B:

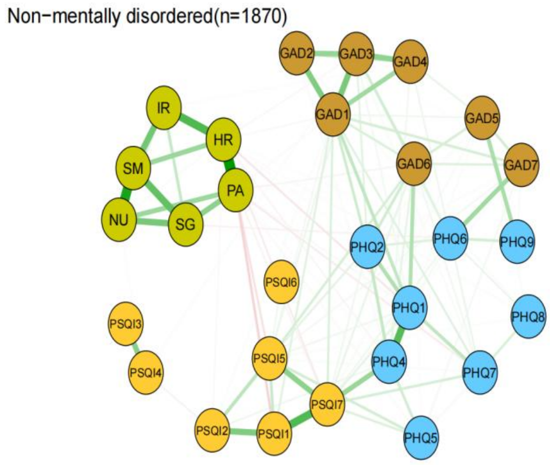

C:

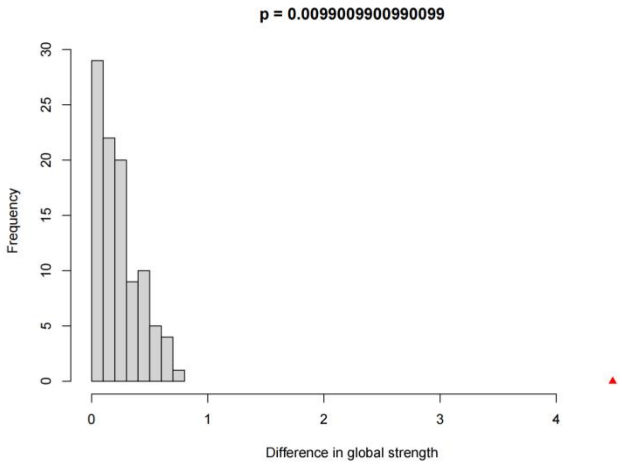

D:

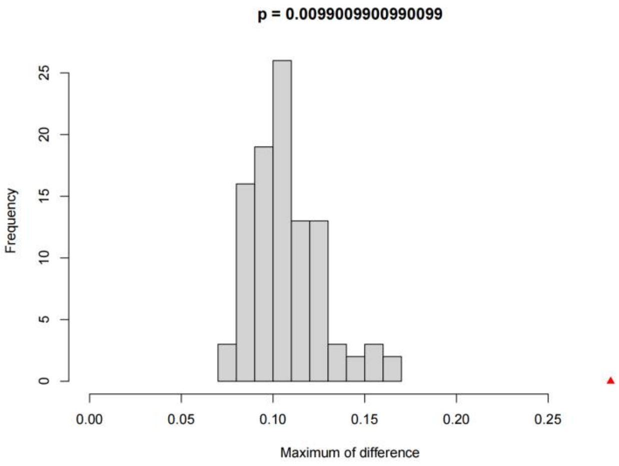

Note: (A) Estimated network model in mentally disordered (n = 2026). (B) Estimated network model in non-mentally disordered (n = 1870).

(C) A plot of bootstrap value of the difference in global strength. The difference was significant (global strength among mentally disordered: 13.21; among non-mentally disordered: 8.72; $p = 0.01$).

(D) A plot of bootstrap value of the difference in network structure. The difference was significant ($M = 0.28$, $p = 0.01$). Invariance in edge weights was tested using the permutation test, generating sets of p values for each edge-edge comparison. Bonferroni-Holm corrected p values were all > 0.05.
